# 4521 Collecting, Interpreting and Utilizing Retrospective Clinical Data from Data Warehouses

**DOI:** 10.1017/cts.2020.172

**Published:** 2020-07-29

**Authors:** Brandon Joseph Sonn, Andrew Monte

**Affiliations:** 1 University of Colorado at Denver

## Abstract

OBJECTIVES/GOALS: Utilizing clinical electronic health record (eHR) data pulled *en masse* from data warehouses provides unique challenges when applying it to retrospective studies. Use of this data in conjunction with metabolomic and genomic results to predict response to lisinopril or ondansetron has been completed. METHODS/STUDY POPULATION: Study population consists of >2000 subjects recruited from the Emergency Medicine Specimen Bank at University of Colorado Denver (UCD). All patients presenting to the emergency department are approached to participate which significantly increases demographic diversity of our study populations. Clinical data is pulled from Health Data Compass (data warehouse at UCD that collects all electronic health record (EHR) data to be able to deliver de-identified). Effectiveness of lisinopril and ondansetron were investigated using metabolomic data collected via ultra-high performance liquid chromatography mass spectrometry and genomic data from Illumina chip technology to find relevant correlations. RESULTS/ANTICIPATED RESULTS: Obtaining retrospective clinical data from data warehouses comes with significant challenges to be addressed. Verifying all clinical variables from patient EHRs is a crucial step that requires extensive quality control steps. As well, ensuring data validity, appropriateness of data points pulled as relate to the study criteria and identifying alternate EHR data points is needed. Chart review is a critical step necessary to surmount these challenges. Additionally, use of retrospective EHR data often necessitates the development of novel definitions of clinical effectiveness that can be abstracted from the EHR– such as how to determine decrease in nausea without a visual analogue scale. DISCUSSION/SIGNIFICANCE OF IMPACT: Utilizing data warehouses to deliver EHR data provides a valuable tool for completing retrospective precision medicine projects. The validation of definitions for clinical outcomes identifiable retrospectively are necessary and provide novel guidance for future studies.

